# Cooperation in Countering Artemisinin Resistance in Africa: Learning from COVID-19

**DOI:** 10.4269/ajtmh.22-0148

**Published:** 2022-04-12

**Authors:** Philip J. Rosenthal, Anders Björkman, Mehul Dhorda, Abdoulaye Djimde, Arjen M. Dondorp, Oumar Gaye, Philippe J. Guerin, Elizabeth Juma, Dominic P. Kwiatkowski, Laura Merson, Francine Ntoumi, Ric N. Price, Jaishree Raman, David S. Roos, Feiko ter Kuile, Halidou Tinto, Sheena S. Tomko, Nicholas J. White, Karen I. Barnes

**Affiliations:** ^1^ University of California, San Francisco, California;; ^2^ Malaria Group, University of Karolinska Institutet, Stockholm, Sweden;; ^3^ Mahidol Oxford Tropical Medicine Research Unit, Bangkok, Thailand;; ^4^ Malaria Research and Training Centre, University of Science, Techniques and Technologies, Bamako, Mali;; ^5^ Department of Medical Parasitology, Faculty of Medicine, Pharmacy and Dentistry, L’Université Cheikh Anta Diop, Dakar, Senegal;; ^6^ Centre for Tropical Medicine and Global Health, Nuffield Department of Medicine, University of Oxford, Oxford, United Kingdom;; ^7^ WorldWide Antimalarial Resistance Network and Infectious Diseases Data Observatory, Oxford University, Oxford, United Kingdom;; ^8^ World Health Organization African Regional Office, Accra, Ghana;; ^9^ Wellcome Sanger Institute, Hinxton, United Kingdom;; ^10^ Fondation Congolaise pour la Recherche Médicale, Brazzaville, Republic of the Congo;; ^11^ Institute for Tropical Medicine, University of Tübingen, Tübingen, Germany;; ^12^ Global and Tropical Health Division, Menzies School of Health Research, Charles Darwin University, Darwin, NT, Australia;; ^13^ South African National Institute for Communicable Diseases, Johannesburg, South Africa;; ^14^ Wits Research Institute for Malaria, School of Pathology, University of Witwatersrand, Johannesburg, South Africa;; ^15^ University of Pennsylvania, Philadelphia, Pennsylvania;; ^16^ Department of Clinical Sciences, Liverpool School of Tropical Medicine, Liverpool, United Kingdom;; ^17^ Kenya Medical Research Institute, Centre for Global Health Research, Kisumu, Kenya;; ^18^ Institut de Recherche en Sciences de la Santé, Nanoro, Burkina Faso;; ^19^ Division of Clinical Pharmacology, Department of Medicine, University of Cape Town, Cape Town, South Africa;; ^20^ WorldWide Antimalarial Resistance Network, Pharmacology Scientific Module, Department of Medicine, University of Cape Town, Cape Town, South Africa

The emergence of *Plasmodium falciparum* parasites with delayed clearance after treatment with artemisinins (artemisinin resistance), first reported in the Greater Mekong Subregion about 15 years ago,^1^^,^^2^ threatens loss of our most important drugs for treating malaria. The subsequent spread and evolution of artemisinin resistance, coupled with the acquisition of resistance to artemisinin-based combination therapy (ACT) partner drugs, have led to high rates of ACT treatment failure in Southeast Asia.^3^ Artemisinin resistance is causally associated with mutations in the propeller domain of the *P. falciparum* Kelch protein (K13) on a suitable genetic background.^4^ Although resistance to artemisinins and partner drugs poses a significant threat to the efficacy of first-line ACTs, its impact in Southeast Asia has been tempered by the relatively low malaria burden and substantial investments in improved malaria control in this region.^5^

However, in sub-Saharan Africa, which bears more than 90% of the world’s malaria burden, progress in malaria control has stalled in recent years, with gains achieved since the turn of the century partially reversed during the COVID-19 pandemic.^5^^–^^7^ In this context, the recent de novo emergence of artemisinin resistance in Africa is of enormous concern. The K13 561H mutation in Rwanda and the 469Y and 675V mutations in Uganda have been documented in 20% or more of symptomatic *P. falciparum* infections in parts of these countries.^8^^–^^13^ All three mutations were previously associated with artemisinin resistance in Southeast Asia, and they have now been associated with resistance both clinically (delayed parasite clearance after therapy) and in vitro (greater survival after artemisinin exposure) in Africa.^10^^–^^13^ Independent emergences of artemisinin-resistant parasites at multiple sites likely reflects strong selective pressure from the widespread use of artemisinins, as previously seen in Southeast Asia. Thus, the artemisinin resistance recently seen in Rwanda and Uganda is likely just the “tip of the spear,” with its emergence and/or spread likely to have occurred already, or soon to occur widely across endemic countries in sub-Saharan Africa.^14^^,^^15^ There is not yet evidence that K13 mutations have been associated with loss of ACT treatment efficacy in Africa. However, in Southeast Asia, the rise in artemisinin resistance was followed by the emergence and spread of resistance to ACT partner drugs (amodiaquine, mefloquine, and piperaquine) and frequent ACT treatment failures.^16^^–^^20^ A similar trajectory in sub-Saharan Africa—in particular with loss of lumefantrine, the most widely used ACT partner drug—would jeopardize the treatment of hundreds of millions of patients each year, likely leading to marked increases in malaria morbidity and mortality.^21^

It is imperative that we act promptly to detect and stem the tide of resistance to artemisinins and ACT partner drugs in Africa. This will require bold leadership and timely evidence-based implementation of changes in malaria treatment and prevention policies. Enhanced surveillance for antimalarial resistance is essential now, but most countries in sub-Saharan Africa only conduct therapeutic efficacy studies every few years, and at only a few sites, and molecular studies to detect molecular markers of resistance are not widely used. This situation risks a delay in the detection of clinically significant artemisinin (and partner drug) resistance until it has become established, increasing treatment failure rates and fueling malaria transmission.

When resistance is confirmed, new strategies to mitigate the spread and consequences of drug resistance must be considered promptly, including rotating multiple first-line treatments, use of triple ACTs (containing two artemisinin partner drugs), and augmenting therapy with single low-dose primaquine to prevent parasite transmission (as currently recommended by the WHO).^22^^–^^24^ These considerations are critical, as new antimalarial combination regimens that do not rely on artemisinins are not expected to be generally available within the next 5 years. Considering the need for increased malaria surveillance and extensive study of new control strategies, and to accelerate malaria elimination, sub-Saharan Africa now needs levels of investment similar to, or greater than, those allocated in recent years to tackle artemisinin resistance in Southeast Asia.

Drug-resistant malaria is a critical public health emergency on a global scale. In sub-Saharan Africa, policy change, and its implementation in particular, has been far too slow in the past, exemplified by the notoriously delayed replacement of failing first-line regimens (chloroquine and then sulfadoxine–pyrimethamine) with ACTs.^21^ This delay was driven as much by financing concerns as hesitancy regarding the strength of the evidence of antimalarial monotherapy resistance. To facilitate rapid policy change and prevent an unnecessary rise in malaria morbidity and mortality, there is an urgent need to characterize the extent and severity of artemisinin and partner drug resistance on the continent. Information must be shared promptly and equitably with local and regional policymakers, the WHO, and the scientific community to help inform public health responses and related research. Many funders and publishers now rightly insist that academic groups make individual participant data accessible, especially in the context of public health emergencies when this information is required urgently to inform public health responses. The same should be required of other organizations that generate these critical data. We urge the malaria scientific community to break from academic “business as usual” and embrace prompt generation and sharing of data concerning antimalarial drug resistance, building on recent experience with the widespread sharing of global SARS-CoV-2 surveillance data.

Traditional scientific processes generally work well to facilitate knowledge generation and advance public health. Incentives to be the first to publish high-impact papers, and competition among academic groups, encourage prompt collection, analysis, and sharing of information in the scientific literature. However, this process is slow, delaying communication of important results to key stakeholders. Peer-reviewed manuscripts on artemisinin resistance are often outdated upon publication; for example, the median time between artemisinin resistance data collection in Southeast Asia and publication was almost 4 years (range, 1–25 years).^25^ The ongoing COVID-19 pandemic has demonstrated clearly that sharing results can be faster and more open, with obvious benefits to public health.

Data-sharing initiatives have shown the power of broad global collaboration to accelerate the understanding and mitigation of novel threats, while simultaneously ensuring appropriate confidentiality and equitably recognizing data providers as global partners and scientific contributors.^26^^–^^28^ The COVID-19 response has also highlighted the importance of sharing research results early through open-access pre-print platforms. Although there are risks associated with these platforms, including potential promotion of confounded and/or poor-quality studies,^29^ the benefits have been immense, facilitating the dissemination of evidence for public health measures, effective vaccines, and antiviral regimens at an unprecedented speed. The COVID-19 pandemic has also shown that the global community can unite to inform and optimize public health interventions when the need is great. A similarly bold approach is well within the grasp of the researchers, organizations, and governments grappling with the threats of antimalarial drug resistance.

In recognition of the advancing public health emergency of artemisinin resistance in sub-Saharan Africa, we propose the following actions to facilitate prompt collection, collation, interpretation, and dissemination of data to inform rapid and effective public health responses.

First, surveillance for artemisinin resistance in sub-Saharan Africa should be strengthened, including increased technical and financial investment, and policies to support the rapid generation and dissemination of surveillance data. Such surveillance should include molecular and parasitological characterization of isolates collected in endemic countries across the continent, clinical evaluations of responses to artemisinins, and studies of the therapeutic efficacy of ACTs, all based on standardized protocols and procedures. As seen with the COVID-19 pandemic, facilitation of access to sequencing facilities through regional or international initiatives is helpful, as many African countries do not yet have this capacity.

Second, all investigators should share results and disseminate key findings rapidly through regional networks, pre-publication platforms, presentation at scientific meetings, and peer-reviewed publication. Peer-reviewed journals should expand their capacity for fast-track publication ahead of print for manuscripts reporting evidence of immediate public health importance, such as clinically significant artemisinin resistance.

Third, all investigators should share de-identified individual patient data from antimalarial therapeutic efficacy studies with local and regional policymakers, the WHO, and the scientific community.

Fourth, platforms for data access and dissemination should be used and enhanced, including linkage between parasite genotype, phenotype, and clinical data. Several established platforms including ClinEpiDB, MalariaGEN, PlasmoDB, WHO Malaria Threat Maps, and the WorldWide Antimalarial Resistance Network (or WWARN) offer expertise in data collation and analysis, and tools to enhance analysis and dissemination of results.^30^^–^^34^

This is a call for cooperation and transparency across the malaria research community, national malaria control programs, the WHO, and international funding bodies, with the goal of preventing a disastrous reversal in the substantial gains made in combatting malaria in sub-Saharan Africa since the turn of the century. Action is needed now. We call on the malaria community to learn from recent COVID-19 pandemic experiences and facilitate prompt, open communication of results, and responsible sharing of data on artemisinin and partner drug resistance from Africa, where artemisinin resistance is now emerging.
